# Launching the Israel Journal of Health Policy Research: Why a new journal? Why now? Why open access?

**DOI:** 10.1186/2045-4015-1-1

**Published:** 2012-01-30

**Authors:** Bruce Rosen, Avi Israeli

**Affiliations:** 1 Myers-JDC-Brookdale Institute, POB 3886, Jerusalem 91037 Israel; 2 Hadassah-Hebrew University Medical Center, POB 12000 Jerusalem 91120 Israel

## Abstract

The *Israel Journal of Health Policy Research *(*IJHPR*) is a new, open access journal. *IJHPR *seeks to promote intensive intellectual interactions among scholars and practitioners from Israel and other countries regarding all aspects of health policy, with particular attention to Israel. The ultimate aim of these interactions is to contribute to the development of health policy in Israel, and also to foster wider communication between health scientists and policy analysts in Israel and their colleagues around the world. This inaugural editorial provides an overview of the new journal's rationale and its key features.

## Editorial

After the many months of preparation, we are excited that IJHPR is now being launched. We are pleased to have this opportunity to share with you some of our goals for the new journal, along with its rationale and key features.

## Journal objectives

As indicated by our official Aims and Scope statement on the journal's website, the *Israel Journal of Health Policy Research (IJHPR) *seeks to promote intensive intellectual interactions among scholars and practitioners from Israel and other countries regarding all aspects of health policy, with particular attention to Israel. These include policy-relevant contributions from any country in fields ranging from health services research, public health, health promotion, health economics, health care management, and the ethics, sociology, and political science of health care, so long as these contributions draw implications for health and healthcare policy in Israel. The ultimate aim of these intellectual interactions is to contribute to the development of health policy in Israel and to foster wider communication between health scientists and policy analysts in Israel and their colleagues around the world.

## The Israel National Institute for Health Policy - the journal's parent organization

To understand the objectives of our journal, readers also need to know a bit about its parent organization. The *Israel Journal of Health Policy Research *is the official publication of The Israel National Institute for Health Policy and Health Services Research. The Israel National Institute is an independent organization whose activities include promoting and funding health services research as well as organizing various forums for the dissemination of research and discussions of current policy issues. In particular, it is worth noting that the National Institute manages the competitive grants process for allocating the monies set aside for health services research by Israel's National Health Insurance Law (1995). Partly as a result of this set-aside and the competitive process that governs the distribution of funds, Israel has a relatively large and vigorous health services research community, with dozens of major research projects underway at any point in time involving researchers from a wide variety of academic and non-university organizations.

## The motivation for launching a new journal

Many Israeli scholars already publish extensively in a wide variety of established international journals in their own fields of expertise, and these Israeli scholars are accustomed to writing about research done in Israel in a way that is relevant to an international audience. Thus, the launching of this new journal was *not *motivated by an absence of vehicles for disseminating research about Israel.

Still, we - the editors and the National Institute - felt the need for a new journal; one which would have Israeli health care as its primary focus. We felt this way for several reasons: First, we wanted to promote a new **interdisciplinary synergy **between studies of different aspects of Israeli health care where the results of studies done by scholars in a number of health-relevant fields would be gathered in the same publication. These opportunities for synergy are often lost when, for example, Israelis researching primary care publish only in journals dedicated to the international study of primary care, and Israelis researching health information systems publish only in journals focused on their own topical area. Some opportunities for synergy exist in various research conferences and policy meetings organized by the National Institute and others, but these primarily oral interactions do not have the benefit of the depth provided by journal articles and buttressed by a vigorous peer review process.

Second, we wanted to create a journal in which the **publication criteria **simultaneously include the contribution to both health systems around the world and to Israeli health care. Many international journals with a disciplinary or topical focus encourage authors to emphasize the universal messages that emanate from their research. This is of course not only valid but also praiseworthy. At the same time, the policy implications for individual countries (in our case Israel) often are lost in the process. We believe that major studies can generate several different articles, some of which emphasize the contribution to a disciplinary field and others that emphasize the contributions to policy - both national and international. IJHPR will clearly focus on the latter.

Moreover, IJHPR is the only health policy journal where contribution to Israeli health policy constitutes a major publication criterion. In such articles, we will encourage the discussion of Israel-specific policy considerations within the context of the Israeli health policy development process. At the same time, we will encourage contributions (and commentaries) that will highlight the congruence or similarities of policy making in Israel in comparison with policy development in other parts of the world.

Third, IJHPR provides us with an opportunity to **focus attention on specific areas of particular interest to Israel**. Some of these will be areas in which Israel has important accomplishments in the field and/or research. These include the use of electronic health records, technology assessment/priority setting, quality improvement in the managed care environment, and the health system response to national emergencies. Others will be areas in which Israel is grappling with challenges similar to those in many other countries; these would include, for example, efforts to reduce inequalities in health and better serve various vulnerable and minority populations. Through IJHPR we hope to bring together a variety of disciplinary perspectives, from a number of countries, on these areas of special interest.

Fourth, we are interested in using IJHPR as a vehicle for **engaging scholars from around the world **in the health policy development process in Israel. We plan to do so using several mechanisms, some of which will be unique to this journal. These include:

• Publishing, alongside each article focused on Israeli health care, a **brief commentary by a leading international scholar**. These commentaries will explore the international significance of the Israeli study and/or provide an international perspective on the study's findings about Israel and their policy implications.

• The establishment of an **editorial board including leading thinkers from both Israel and other countries**. We are particularly pleased about the participation of several past editors, and current editorial board members from leading international health policy journals. This will contribute to the rigor of the review process and the quality of the published articles. As a side benefit, it may also help several international journals become more acquainted with, and more interested in, developments in Israeli health care and the capabilities of the Israeli research community.

• **Encouraging collaborations between Israelis and non-Israelis **as joint authors of either original research articles or integrative/review articles. These might include articles comparing two or more health systems, articles exploring the international relevance of an Israeli development, articles exploring the relevance to Israel of international developments, etc.

## The cosmopolitan nature of Israeli health care

Learning from other countries has always been a central feature of Israeli health care - at the level of individual professionals as well as at programmatic and policy levels. Most young Israeli physicians interested in pursuing subspecialty training do so abroad. Israeli academics are encouraged financially to spend their sabbaticals at institutions of higher learning in other countries, and to participate in international conferences around the world. Israeli health plans and hospitals regularly seek out new programmatic developments in other countries.

At the policy level, Israelis are also voracious learners and adapters. Dutch precedents influenced the establishment of Israel's capitation financing mechanism and its National Health Insurance system, more generally. Israel's DRG payment system and its health plan quality monitoring system both drew heavily on U.S. initiatives. The UK has been an important model for the regional organization of the public health system and for efforts (to date only partially successful) to decentralize the government hospital system.

In keeping with this tradition, the National Institute has always made cross-national learning a vital component of its activities. Every three years, it organizes a major international health policy conference, with over 500 participants from over 20 countries. In addition, each year, the National Institute organizes 2-3 international workshops on pressing issues in Israeli health policy. Participation typically includes key Israeli health care leaders involved in the issue, along with 3-5 relevant experts from other countries. The workshops usually last two days, with the first day typically dedicated to presentations on the achievements and challenges facing each of the countries represented, and the second day to teasing out the transferable ideas, keeping in mind the substantial cultural and organizational differences among the countries.

These international workshops constitute not only an important potential source of articles for IJHPR, but also inspiration and a model. As those workshops demonstrated, cross-national learning can be particularly effective when it focuses on a specific issue that is a shared high policy priority among several countries. They also highlight the critical importance of a thorough understanding of each system involved, along with an analysis of their commonalities and differences, prior to any efforts to draw lessons across systems. Finally, they demonstrate the value of intense intellectual interactions among experts from different countries. We hope to bring these lessons to bear by encouraging joint articles by Israelis and non-Israelis, commentaries by international experts on articles that focus on Israel, and in articles that explore the relevance to Israel of interesting initiatives in other countries.

## The decision to adopt an online, open access model

Finally, a word about why we have chosen the online, open-access format, the opportunities that this format affords us, and our choice of BioMed Central as the journal's publisher.

Online/open access is the fastest growing segment of the academic journal market, due to its wide reach, low costs, and fast turnaround times. It is particularly suited to the needs of a journal such as ours that seeks to promote international discourse focused on the health system of a specific, relatively small, country. While few libraries are likely to subscribe to a print journal focused on Israeli health care, many researchers and practitioners around the world are likely to be interested in those aspects of Israeli health care that touch upon their own areas of activity. The combination of sophisticated search tools (such as Pubmed and Google) and the online/open access model means that they will be able to easily identify and access any IJHPR article of interest to them. The fact that all the article processing charges will be covered by the Israel National Institute removes any cost barriers for the authors as well. As a result, IJHPR will constitute a free and easy to use communication channel between the generators of the studies and their potential users.

The online/open access format also provides additional opportunities. Readers can provide rapid response comments on articles, so that the publication of an article can serve as the beginning of a discussion rather than as a one-time pronouncement. Information on the number of article downloads is available immediately and on an ongoing basis, giving us the opportunity to see which topics and types of articles generate the greatest reader interest. The online format also makes it possible to use thematic series to focus attention on topics of particular interest. In comparison with their print counterpart, the "special issue", thematic series have the advantage of sustained attention to a topic over time. In addition, they make it possible to avoid the well-known editors' nightmares of special issues being held up by the last article due to be submitted.

In short, we see this endeavor as an attempt to integrate social networking technology for a scholarly purpose, one that can bring the international community of scientists and policy analysts working on salient issues in health and health care into an ongoing "conversation" that follows the rules of academic discourse and the time-honored criteria of scholarly merit, but is open to rapid and free-flowing communication, similar to that of a seminar. We are seeking to make this technology work for us, and for the constituencies we seek to inform through our work in health and health care policy science and practice.

Once we decided to go with the open access format, the choice of BioMed Central as publisher was an easy one. BioMed Central (or BMC for short) is the clear leader in the field, with over 200 Open Access publications. Their recent acquisition by Springer Ltd., the world's second largest Scientific, Technical, and Medical publisher, has provided them with additional expertise and resources. More than any other publisher active in the health care field, BMC understands the potential, and the limitations, of the Open Access model. BMC's size makes it feasible for it to invest in the development of the journals' infrastructures, as witnessed by the recent upgrading of journal websites. We look forward to a long and successful partnership with BMC.

## IJHPR as a partnership between editors, authors, and readers

We similarly look forward to a long and successful partnership with you, our authors and readers. Quite naturally, the journal's first years will be characterized by a fair amount of experimentation, and there are likely to be both successes and mistakes along the way. We are eager to hear your suggestions and feedback, particularly at this formative stage. Please feel free to write to us at editorial@ijhpr.org about anything, anytime.

Happy reading!

## Authors' information

Avi Israeli is the Dr. Julien Rozan Professor of Family Medicine and Health Care at the Hebrew University -- Hadassah Faculty of Medicine; Director of the Department of Health Policy, Health Care Management and Health Economics, Hebrew University -- Hadassah Braun School of Public Health & Community Medicine; Chief Scientist of the Ministry of Health; and co-editor of the IJHPR.

Bruce Rosen is Director of the Smokler Center for Health Policy Research at the Myers-JDC-Brookdale Institute, as well as co-editor of the IJHPR.

